# Machine Learning Prediction of Thermal Properties of PHB/PHBV-Based Materials: A Quantitative Structure–Property Relationship Approach Using an Integrated Polymer Database

**DOI:** 10.3390/polym18131559

**Published:** 2026-06-23

**Authors:** Nikolaos P. Sotiropoulos, Leonidas Mindrinos, Jean-David Peltier, Konstantina V. Filippou, Marianna I. Kotzabasaki, Nikolaos Tsigkas, Chrysanthos Maraveas

**Affiliations:** 1 Department of Natural Resources Development and Agricultural Engineering, Agricultural University of Athens, Iera Odos 75, 11855 Athens, Greece; nikos.sotiropoulos@aua.gr (N.P.S.); leonidas.mindrinos@aua.gr (L.M.); mariannakotz@aua.gr (M.I.K.);; 2 CETEC Centro Tecnológico del Calzado y del Plástico de la Región de Murcia, Polígono Industrial Las Salinas, Avenida Europa, 2–3, 30840 Murcia, Spain

**Keywords:** Poly(3-hydroxybutyrate), Poly(3-hydroxybutyrate-co-3-hydroxyvalerate), thermal properties, machine learning, quantitative structure–activity relationship models, polymer informatics

## Abstract

Bio-based and biodegradable polymers such as short-chain-length (scl) poly(3-hydroxybutyrate) (PHB) and poly(3-hydroxybutyrate-co-3-hydroxyvalerate) (PHBV) are widely adopted in diverse areas such as healthcare, manufacturing, and packaging. However, high production costs and the complexity of tailoring their thermal properties, such as glass transition temperature (Tg), melting temperature (Tm), and crystallization temperature (Tc), hinder further adoption. The current study reported on the development of a raw dataset of PHB and PHBV materials compiled from 572 instances collected from the literature (558 instances) and in-house experiments (14 instances). The dataset encompassed compositional physicochemical parameters, molecular features, and corresponding thermal characteristics. After assessing data quality and filtering for completeness and available features, curated datasets were created for machine learning (ML) analysis. Two ML models, Random Forest (RF) and eXtreme Gradient Boosting (XGBoost), were utilized to predict values of Tg, Tc, and Tm using feature engineering methods that integrated chemistry-based descriptors with polymer-specific and experimental variables. The predictive performance of the models was systematically investigated using different combinations of input features to identify the most informative descriptor sets for each target property. The best-performing models were obtained using 118 data points for Tg and Tm and 201 data points for Tc, achieving R^2^ values of 0.77, 0.76, and 0.82 for Tg, Tc, and Tm, respectively. Despite the reliable prediction of the thermal properties of scl-PHAs, the main limitations of the study were the relatively small dataset size for certain targets and incomplete or missing reporting of experimental conditions in the literature sources, which may introduce variability in the compiled data. The findings implied that curated polymer datasets and interpretable ML models can support the rational design of sustainable polymers with tailored properties for specific applications.

## 1. Introduction

Conventional plastics, based on their durability, versatility, and low costs of production, are widely applied in different industries such as packaging, healthcare, and manufacturing. However, they also directly contribute to environmental pollution, as plastic waste is discarded into oceans, rivers, and landfills [1]. Modern methods to reduce pollution, such as recycling, are ineffective based on the small fraction of waste that they eliminate [2]. As a result, a significant proportion of plastic waste continues to pollute the environment as it takes decades to degrade into microplastics (MPs) and nanoplastics (NPs), risking ecosystems and human health [3]. Due to inadequate plastic waste management processes, there is an urgent need to transition to a circular economy (CE), ensuring a sustainable strategic framework governing the use of plastics.

The redesign of production, use, and end-of-life management of plastics is a promising pathway to eradicate plastic pollution and its negative environmental impacts [4]. Additionally, developing and adopting sustainable, bio-based, and biodegradable plastic products (BBpPs) is an effective alternative [5,6]. Polyhydroxyalkanoates (PHAs), a family of bio-based and bio-renewable aliphatic polyesters, are an example of a BBpP that biodegrades in different natural environments and are biosynthesized by microorganisms through fermentation under nutrient-limiting conditions [7,8]. Bacteria synthesize PHAs intracellularly as carbon and energy storage polymers composed of hydroxyalkanoate (HA) units. However, a core challenge of PHAs is their high production costs [9,10,11]. As a result, this limits their use in commercial settings.

PHAs are also categorized based on the length of the alkyl side chain in their repeating units into three groups. First, there are scl-PHAs with three to five carbon atoms, followed by medium-chain-length PHAs (mcl-PHAs) with six to 14 carbon atoms. Third, there are long-chain-length PHAs (lcl-PHAs; 15 or more carbon atoms) [12]. Additionally, the physicochemical and thermal behavior of PHAs is governed by factors such as average molecular weight, monomer composition, side chain length, and crystallinity, which can be modified to tailor the performance of the material. Further, the ability to tune the properties of PHAs, along with their biodegradation and biocompatibility in natural environments, is a core feature that positions PHAs as strong candidates that are suitable alternatives to traditional plastics derived from petroleum [11]. In this context, PHAs can be adopted to support the development of a circular bioeconomy.

Further insights from the literature reveal that PHB and its copolymer PHBV are the most widely studied scl-PHAs. These polymers (PHB, PHBV) have side chains attached at the third carbon atom of the backbone and exhibit relatively high crystallinity and melting temperatures. The integration of comonomers such as 3-hydroxyvalerate into PHB also disrupts the regularity of the crystals and further reduces the melting point while also introducing changes in chain mobility [4]. Additionally, PHB demonstrates improved thermal processability in terms of lower Tm, Tc, and Tg due to its copolymer (PHBV), leading to elastic and flexible texture at normal temperature while guaranteeing easier processing.

The blending of scl-PHAs with mcl-PHAs or the integration of additives such as plasticizers also modifies intermolecular interactions and crystallinity, enhancing thermal behavior. While these approaches enhance performance and processability, the resulting structure–property associations are often nonlinear and strongly coupled, leading to difficulty in making rational design using conventional trial-and-error methods. In contrast with scl-PHAs, mcl-PHAs also contain longer side chains that hinder chain packing, thereby leading to lower crystallinity, lower melting points, and elastomeric behavior [13,14]. The distinct structural differences lead to distinct mechanisms of crystallization and thermal transitions, revealing the chemically and physically unique polymer class of scl-PHAs. Additionally, the thermal qualities of scl-PHAs, especially Tg, Tm, and Tc, are highly sensitive to molecular structure and formulation. PHB, the most widely industrialized PHA, is also brittle due to its high crystallinity, poor mechanical properties, and low thermal stability because its melting temperature (Tm) is close to its degradation temperature (Tdeg), making thermal processing (e.g., extrusion, injection molding) challenging [15,16].

Despite the unique advantageous properties of PHAs (high crystallinity and melting temperatures and easier processing), they are inherently limited by factors such as brittleness, narrow processing windows, sensitivity of thermal properties to small compositional changes, and high production costs [15]. Such challenges are highly pronounced for scl-PHAs, where modest variations in comonomer content, blend composition, or additive concentration can lead to significant shifts in Tg, Tm, and Tc [14]. Consequently, predictive tools for the efficient linkage of formulation parameters to thermal properties are essential to accelerate the development of application-ready scl-PHA- and PHB-based materials with targeted properties, reducing experimental costs and time. To predict polymer properties and guide the design of the materials, ML methods and polymer informatics have also emerged as a promising alternative [17,18,19]. However, the success of ML models is constrained by the lack of high-quality and chemically consistent datasets. For PHAs, the availability of high-quality datasets is yet to be met despite large polymer databases such as Polymer Genome and PoLyInfo, in which PHAs represent only a small fraction of entries and thermal property data are often incomplete or inconsistently reported [20,21].

Moreover, clear differences do not emerge between scl- and mcl-PHAs in these databases, while they fail to account for formulation effects such as additive incorporation and blending. Currently, only a limited number of ML studies have addressed thermal property prediction within the PHA family. Previous studies have demonstrated the feasibility of predicting Tg or Tm for PHA homopolymers and copolymers based on small, literature-curated datasets, while more recent multitask deep learning models have included PHAs as a minor subset within broad collections of polymers. Such approaches either rely on heterogeneous PHA datasets, which obscure scl-specific physicochemical trends, or prioritize general polymer screening rather than focused modeling of scl-PHA formulations [22,23,24,25,26]. As such, a gap exists in the lack of high-quality datasets related to PHAs that address formulation effects related to blending and additive incorporation.

The aim of the current study was to develop a curated and standardized dataset of PHB and PHBV materials compiled from the literature and in-house experiments and apply ML models to predict their thermal features. The objectives in the study were:

- To curate a structured data library of PHB/PHBV-based materials incorporating various additives and building blocks from the literature and in-house experiments.
- To implement XGBoost and Random Forests to predict the thermal features of scl-PHAs from the curated dataset.
- To critically investigate the restrictions of the ML models and the curated database, thereby outlining future research directions.

The novelty of this study was its presentation of a curated dataset dedicated exclusively to scl-PHAs with side chains at the 3-position of the polymer backbone, including PHB, PHBV formulations, additive-containing systems, and blends with mcl-PHAs, unlike broad polymer databases such as Polymer Genome and PoLyInfo, in which PHAs represent only a small subset of entries, and unlike previous PHA datasets that primarily focus on homopolymers and copolymers, allowing the relationship between formulation composition and thermal behavior to be systematically explored. Experimental Tg, Tc, and Tm values, weight-average molecular weight (Mw), number-average molecular weight (Mn), polydispersity index (PDI), and compositional information were systematically obtained from the literature and critically curated to ensure data consistency and reliability. Narrowing the scope to only these specific materials ensured that a thoroughly curated dataset could be constructed and aided the development of predictive models that captured structure–thermal property relationships and additive interactions in Tg, Tc, and Tm, inaccessible in broader PHA datasets. Subsequently, the research aims to establish a foundational data infrastructure for scl-PHA informatics and demonstrate the value of targeted curation for advancing predictive polymer design and optimization of scl-PHA formulations with tailored thermal performance.

## 2. Materials and Methods

### 2.1. Workflow of Model Development

**Figure 1:**
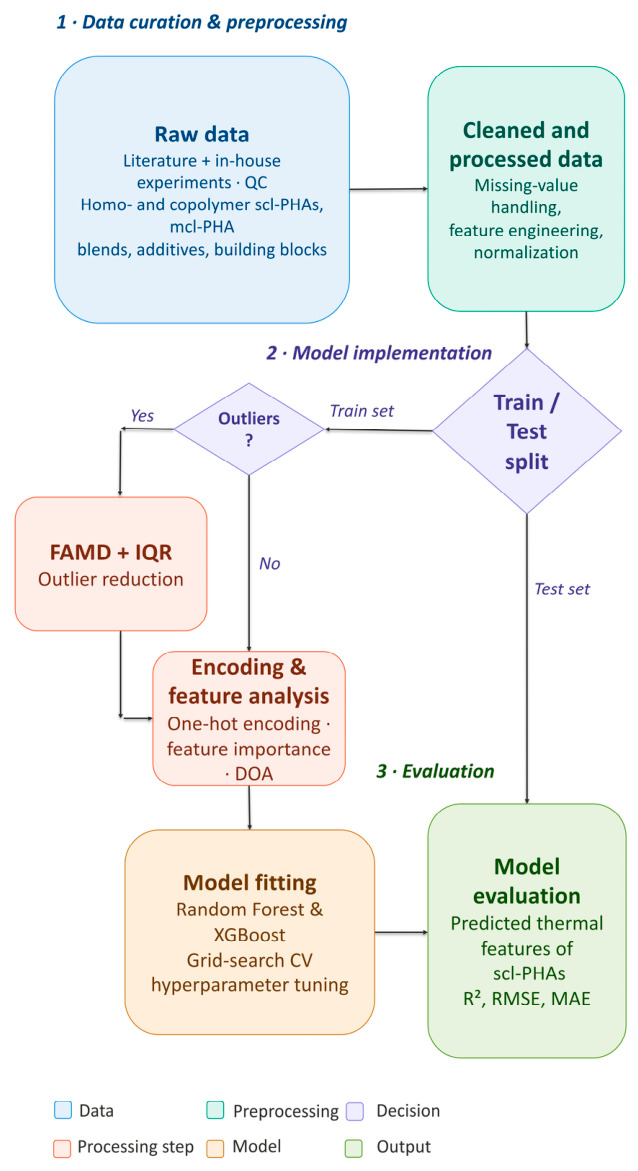
Research workflow of model development.

The present study follows a multi-stage research methodology that integrates literature data analysis, experimental data incorporation, database development, feature engineering, and machine learning modeling. The overall workflow is depicted in Figure 1 and summarized as follows.

In the first stage, a comprehensive data curation process was conducted to assemble a structured dataset comprising polymer composition, molecular characteristics, additive information, and physicochemical and thermal properties. Raw data were manually collected from peer-reviewed literature sources as well as in-house experimental measurements to ensure broad coverage of scl-PHA systems. An initial quality control procedure was applied to remove duplicate entries and resolve inconsistencies in units. This step resulted in a curated and standardized database suitable for downstream analysis.

The second stage focused on data preprocessing and feature engineering. Missing values in key molecular descriptors were handled using physically consistent reconstruction strategies based on established polymer relationships, thereby improving descriptor coverage while preserving chemical meaning. Numerical features were subsequently normalized and transformed to ensure comparability across different scales, while categorical variables related to polymer type, composition, and additives were encoded using one-hot encoding. This stage also included preliminary exploratory analysis to assess feature distributions and potential sources of bias in the dataset.

In the third stage, ML model development was carried out. The dataset was partitioned into training and test subsets using multiple splitting strategies to evaluate robustness. Outlier detection was applied exclusively to the training data to prevent information leakage. Random Forest (RF) and XGBoost models were trained using cross-validated hyperparameter optimization to balance model complexity and generalization [27,28]. Feature importance analysis was performed to identify the most influential descriptors governing thermal behavior, and a Domain of Applicability (DoA) analysis was implemented to define reliable prediction ranges in terms of composition, molecular weight, and additive content. Model evaluation and validation were conducted using multiple complementary metrics and validation strategies. Predictive performance was assessed using the coefficient of determination (R^2^) together with error-based metrics (MAE and RMSE), and uncertainty was quantified through cross-validation variability.

### 2.2. Data Collection from the Literature

#### 2.2.1. Literature Search Strategy

The literature search was undertaken in verified scientific databases such as Scopus, Elsevier, and Springer in alignment with PRISMA 2020 guidelines [29]. The rationale for their selection was that they provide access to diverse peer-reviewed research articles, patents, and conference proceedings, thereby providing valuable scientific insights to address the research objectives.

Keywords were first derived from the research objectives, such as “PHBV,” “PHB,” “thermal,” “lignin,” “ascorbic acid,” “orotic acid,” “maltose,” “thymol,” and “sorbitol.” Boolean operators AND/OR were then adopted to create a search string used in performing a literature search in the individual databases.

The final search string was structured as follows, where the TITLE-ABS-KEY search function from Scopus was used to query the databases:

TITLE-ABS-KEY ((“PHBV” OR “polyhydroxybutyrate-co-valerate” OR “Poly(3-hydroxybutyrate-co-3-hydroxyvalerate)” OR “polyhydroxybutyrate” OR “PHB”) AND (“thermal” OR “rheological”) AND (“lignin” OR “ascorbic acid” OR “orotic acid” OR “theobromine” OR “fructose” OR “glucose” OR “lactose” OR “sucrose” OR “maltose” OR “melezitose” OR “dextran” OR “ascorbyl palmitate” OR “lignins” OR “ammonium quaternary salts” OR “calcium carbonate” OR “epoxidized soybean oil” OR “acetyl tributyl citrate” OR “castor oil” OR “limonene” OR “thymol” OR “starch-based fillers” OR “sorbitol” OR “maltodextrin” OR “hexadecyl 3,5-bis-tert-butyl-4-hydroxybenzoate” OR “PHN” OR “polyhydroxynonanoate” OR “Poly(3-hydroxynonanoate)”)).

Applying the search string resulted in the retrieval of 327 documents.

#### 2.2.2. Study Selection and Screening

**Figure 2:**
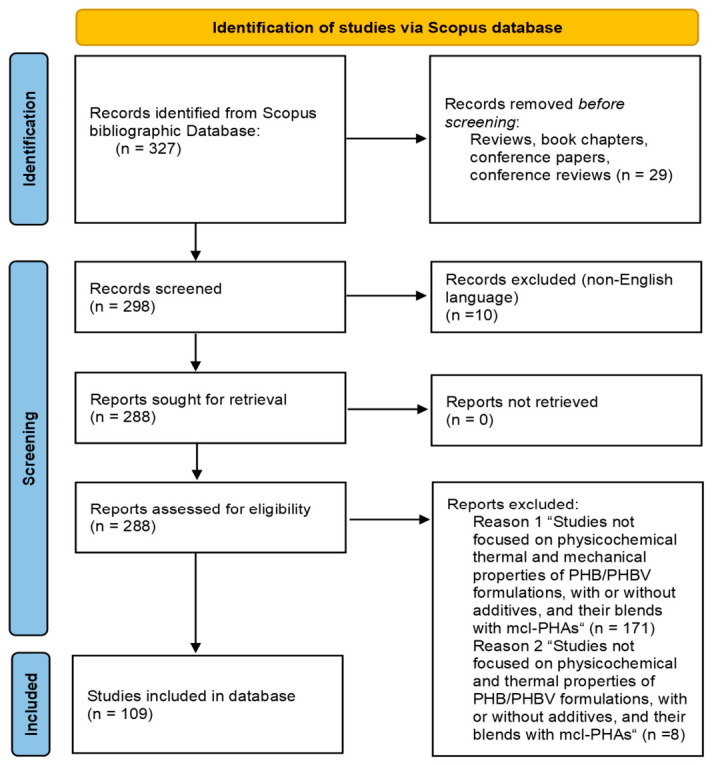
PRISMA 2020 flow diagram illustrating the identification, screening, eligibility, and inclusion of studies for database construction of PHB/PHBV-based materials [29].

Inclusion and exclusion criteria were further defined to facilitate the selection of relevant studies through a screening process based on the PRISMA 2020 guidelines [29] (Figure 2).

Based on the PRISMA 2020 guidelines [29], the research only selected studies whose scope focused on the thermal properties of PHB/PHBV formulations, their compositional information, with or without additives, and their blends with mcl-PHAs. Only original research articles, published in English, were included. There were no restrictions on the publication year, ensuring wide coverage of historical information. However, articles published in non-English languages and review articles were excluded from the study. Subsequently, only 109 studies were included in the final study.

#### 2.2.3. Data Extraction and Synthesis

To extract and synthesize data from the identified 109 studies, a comprehensive examination of each study was undertaken. The data extraction from the literature sources identified the compositional, physicochemical, and thermal properties of the PHB/PHBV compounds for a wide range of molecular weights and the percentages of monomers that constituted them.

**Figure 3:**
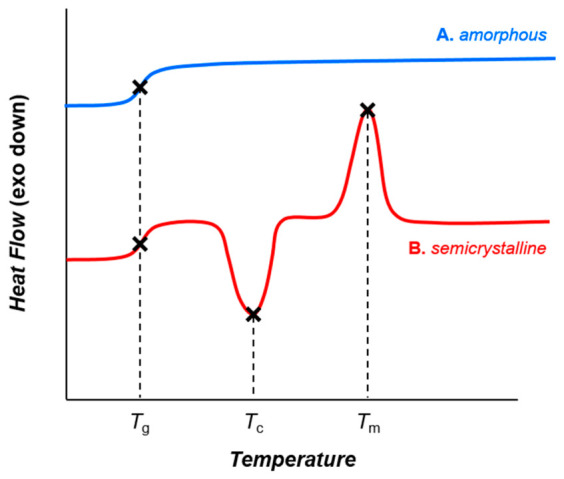
DSC thermogram and thermal transitions for A. amorphous and B. semicrystalline polymers. Reproduced from [30], Wikimedia Commons, 2018.

Further, data was also extracted from the in-house experimental dataset based on existing standards and protocols. First, Differential Scanning Calorimetry (DSC) was adopted to determine Tg, Tm, and Tc, as it directly assesses thermal transitions from differences in heat flow by detecting energy absorbed (endothermic) or released (exothermic) during phase transitions. Next, CETEC experimental protocols were implemented to facilitate the integration of the experimental dataset (14 instances). In cases where thermal properties were not reported but graphs, images, or curves provided useful data, the values were manually extracted using PlotDigitizer. While this approach ensured that all relevant thermal property data, including Tm, Tg, and Tc, were captured, even when only graphical representations were available, it can increase uncertainty and introduce manual errors. Additionally, due to incomplete reporting in the literature, key DSC experimental conditions such as heating/cooling rate, thermal history, and the definition of transition temperatures (onset, midpoint, or peak) were not consistently available and thus could not be incorporated into the dataset. These variations in DSC measurement conditions and methods used across studies can influence the measured thermal transitions and lead to shifts in the reported Tg, Tm, and Tc values, thereby representing an unavoidable source of variability and heterogeneity within the compiled dataset. In cases where sufficient information was available, Tc was taken from the cooling cycle, while Tg and Tm were extracted from the second heating scan to minimize the influence of prior thermal history. A representative DSC diagram for amorphous and semicrystalline polymers is shown below in Figure 3.

#### 2.2.4. Data Curation

The literature and experimental data were curated in a central Excel Spreadsheet with 3 worksheets. All features were standardized to consistent units (SI or SI-consistent where applicable) to ensure comparability across datasets prior to model development. Abbreviations from the dataset were also documented in a fourth worksheet. Each worksheet represented a different data category, organized as showcased in Table 1 below.

Each study was also assigned a unique identification number (study ID), and relevant information across each category was entered into a row. If a study included different compositional or experimental details, a separate category (instance) was created for the study. Despite representing different formulations and conditions, these categories retained the study ID of their respective source studies.

#### 2.2.5. Data Integration

The data from the literature (558 instances) and in-house experiments (14 instances) were further integrated into a database, leading to 572 different instances. The study IDs were non-sequential as they were extracted from a larger data library. Table A1 in Appendix A.1 showcases the distribution of instances for each study. The dataset was designed following the Findability, Accessibility, Interoperability, and Reuse (FAIR) data principles, facilitating reproducibility and reuse [31].

Table A2 in Appendix A.2 outlines the input (feature) and output (target) variables included for the construction of the scl-PHAs-based materials data library, which includes 25 numerical and 12 categorical variables. Each variable is described based on its definition, measurement unit, value range, and type (categorical or numerical). The full curated raw data library for the scl-PHAs-based formulations, including all variables across the three worksheets, was uploaded to the Agricultural University of Athens (AUA) Zenodo repository [32].

### 2.3. Data Collection from In-House Experiments

#### 2.3.1. Experimental Data Acquisition Methodology

Following the collection of data from the literature, the second phase involved conducting in-house experiments to extract and characterize PHBV, as described in the subsequent subsections, resulting in the addition of 14 new instances to the dataset. The experimental values introduced into the database were single measurements.

##### PHBV Extraction

First, purified PHBV was recovered from the PHBV-rich biomass produced at the CetecBio PHBV pilot plant. Dried PHBV-rich biomass (about 700 g) was ground using a RETSCH cutting mill SM300 mounted with a 6 mm sieve. The ground PHBV-rich biomass was Soxhlet-extracted for 12 h with 1,3-dioxolane (4 L), and the extract was concentrated until the formation of a brown gel in a rotary evaporator. Methanol at −18 °C (1 L) was added to the residue, and the solution was agitated to precipitate the PHBV. The precipitate was then washed 3 times with methanol (500 mL) or until the filtrate was colorless. The recovered PHBV was then dried in the oven at 60 °C overnight, and the yield was recorded.

##### Gel Permeation Chromatography (GPC)

The GPC data were recorded on an Agilent Infinity II instrument equipped with differential refractive index (DRI), viscometry (VS), and light scatter (LS) detectors. The system was equipped with 2 × Agilent PLGel Mixed C columns (300 × 7.5 mm) and an Agilent 5 µm PLGel Guard column. Agilent poly(methylmethacrylate) (PMMA) EasiVials were used to create a third-order conventional calibration from DRI data between 1,795,000 and 535 g mol^−1^. The mobile phase was CHCl_3_, run at a flow rate of 1 mL min^−1^ at 30 °C. All sample analyses (number-average molar mass (Mn), weight-average molar mass (Mw), and polydispersity index (Mw/Mn)) were carried out using Agilent GPC/SEC software.

##### Differential Scanning Calorimetry (DSC)

DSC was performed on the purified PHBV (10 ± 5 mg using TzeroTM aluminum pans and a TA Instrument, DSC 2500, with refrigerated cooling under nitrogen (50 mL/min) based on the international standard UNE-EN ISO 11357-1 [33]. The DSC equipment was calibrated using indium and sapphire standards in adherence with manufacturer protocols. Samples were equilibrated at −70 °C (2 min), then ramped at 10 °C/min to 185 °C (3 min) to remove any thermal history, then cooled to −70 °C at a rate of 10 °C/min (2 min), and the cycle was repeated.

The glass transition temperature (Tg) was determined based on the international standard UNE-EN ISO 11357-2 [34]. The melting temperature (Tm) and melting enthalpy (∆H_f_) were further determined from the inflection point and endothermic peak of the second heating scan. The crystallization temperature (Tc) was based on the exothermic peak observed during the cooling cycle (in the presence of a nucleating agent) or during the second heating cycle (in the absence of a nucleating agent) following the international standard UNE-EN ISO 11357-3 [35].

The degree of crystallinity (Xc) was determined using the following equation:

(1) $$X_{C}=\frac{\Delta H_{f}}{{{\Delta H}^{0}}_{f}}\times 100$$

where ΔH_f_ indicated the melting enthalpy of the sample and ${{\Delta H}^{0}}_{f}$ showed the melting enthalpy of the pure crystalline polymer (146 J/g for PHBV) [36]. Thereafter, the crystallization temperature was determined. Data were analyzed using TA TRIOS v5.1.1 software.

##### Nuclear Magnetic Resonance (NMR) Spectroscopy

PHBV samples were dissolved in deuterated chloroform (CDCl_3_), and 1H-NMR spectra were recorded on an Ascend Bruker 400 MHz spectrometer at room temperature. Spectra were analyzed using MestReNova V14.2.0 software. The molar fraction of the 3 HV unit of the PHBV samples was estimated following the CEN workshop agreement 18155 procedure [37].

#### 2.3.2. Mechanical Testing

Mechanical tests were carried out in a controlled atmosphere at 23 °C and an RH of 48% using a Zwick/Roell Z010 apparatus based on ISO 527 [38]. The preload was 1 N, the testing speed was 50 mm/min, the initial clamping length was 115 mm, and the initial standard stroke length was 50 mm.

#### 2.3.3. Data Preprocessing

In the subsequent phase, the dataset “Worksheet_1_Physicochemical Information,” consisting of 24 columns and 572 rows, and the dataset “Worksheet_2_ Thermal properties”, with 18 columns and 572 rows, were merged based on the key columns “Study_id” and “Instance”. This integration aligned each experiment’s physicochemical properties with the extracted temperature values.

Before the model was developed, a series of preprocessing and feature engineering steps was applied. For samples where the Mw (Mw_PHBV), the number-average molecular weight (Mn_PHBV) and the PDI were not all available or only one of them was not reported, the missing values were recovered following the standard polymer relationship:

(2) $$M_{w}=PDI\times M_{n}$$

In Table 2, Mw values before and after reconstruction are summarized, together with the percentage of filled entries compared to the full dataset of 572 samples (instances). From the perspective of data completeness, Mw achieved the highest coverage in the curated dataset, e.g., 64.2%, after reconstruction, compared to 41.1% for Mn and 40.4% for PDI. However, beyond data availability, Mw was the most relevant molecular weight descriptor for the thermal properties of semicrystalline PHB and PHBV. An explanation was that Mw reflected the length of the polymer chains produced during fermentation for the type of biopolyesters. Longer chains, e.g., higher Mw, resulted in a more entangled, cohesive material with higher Tg and Tm. On the contrary, shorter chains, e.g., lower Mw, behaved more like diluents within the polymer matrix, reducing both Tm and Tc [39]. Furthermore, Mn captured the shorter chain fraction of the distribution and was more sensitive to the presence of oligomers; hence, it was a less stable predictor of bulk thermal behavior. Finally, PDI described the width of the molecular weight distribution that affected the thermal properties.

The original additive type labels showed significant heterogeneity due to differences in terminology. For example, “plasticizer” and “plastisizer” were included as separate labels, as well as several categories with fewer than ten instances. To reduce categorical sparsity and improve model robustness, additive types were grouped into a reduced number of meaningful classes. In this study, this involved a trade-off between data availability and the specific roles of the additives in governing mechanisms such as crystallization. Therefore, the model learnt a generalizable representation of an additive function, in line with established QSPR/QSAR (Quantitative Structure–Activity Relationship) methodologies for categorical feature encoding [40]. This decision also prevented excessive dimensionality from one-hot encoding in relation to the limited dataset size, thereby preserving an appropriate sample-to-feature ratio and reducing the risk of overfitting.

“Plasticizer,” “filler,” “polymer_modifier,” and “stabilizer” were the four defined categories. Each category corresponded to a distinct mechanism of thermal property modification in PHB/PHBV formulations. More specifically, plasticizers increased the fractional free volume and reduced both Tg and Tm by disrupting the chain packing regularity [41]. Fillers, nucleating agents, and reinforcements were also grouped based on their similarity in providing heterogeneous nucleation sites or restricting bulk chain mobility, therefore elevating and modifying crystallinity [42]. Additionally, polymer modifiers changed the structure and interaction of the polymer chain by adding new particles to improve properties, and, finally, stabilizers prevented the polymer chains from breaking when heated, keeping their molecular weight stable during processing [43]. Supplementary material related to category grouping is showcased in Table 3 and Table 4, which illustrate the mapping of original to merged categories for “Additive_type_1” and “Additive_type_2”, respectively.

Missing additive weight fractions were considered as the absence of the corresponding additives. As such, missing values in the additive variables were replaced with zero. Similarly, for samples where the additive was absent, the corresponding additive type was assigned the category “not applicable”. In the final merged dataset, column features with more than 70% missing values and columns containing irrelevant information (e.g., “Additive1_name”, “Temperature_units”) were excluded. The cleaned dataset with completeness percentages of every feature is presented in Table 5.

**Figure 4:**
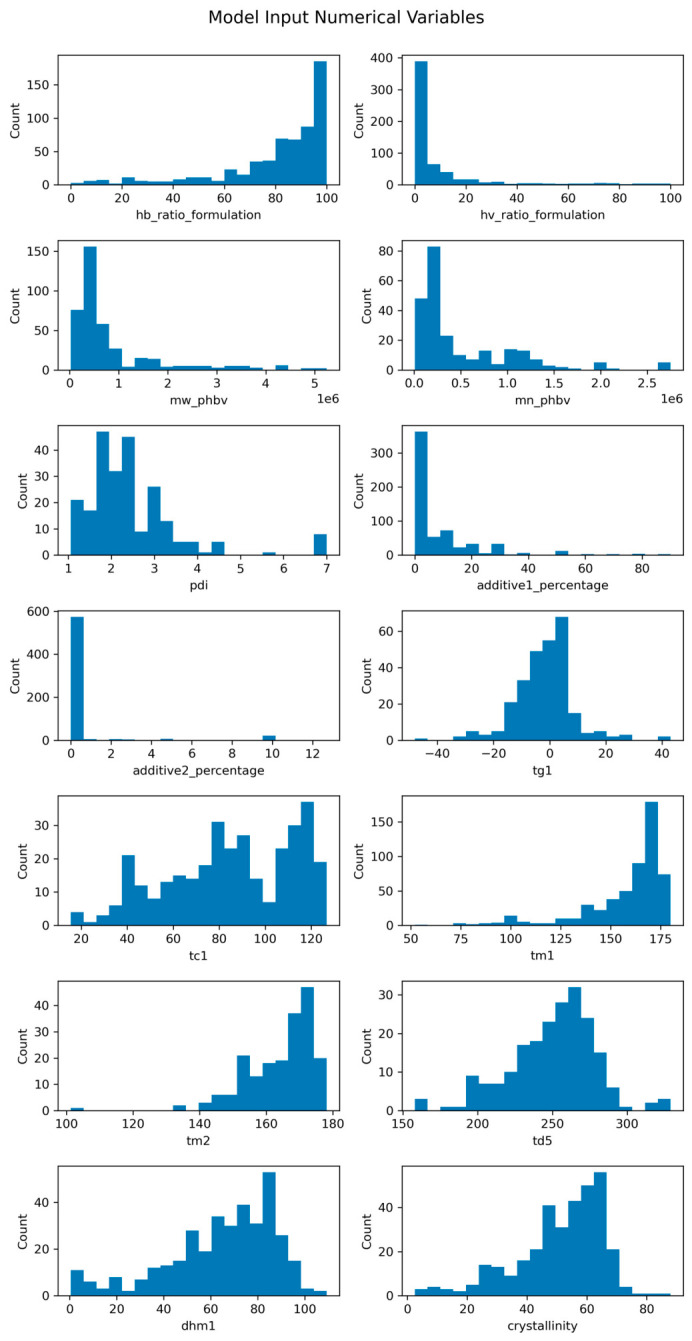
Distribution of model input numerical variables.

**Figure 5:**
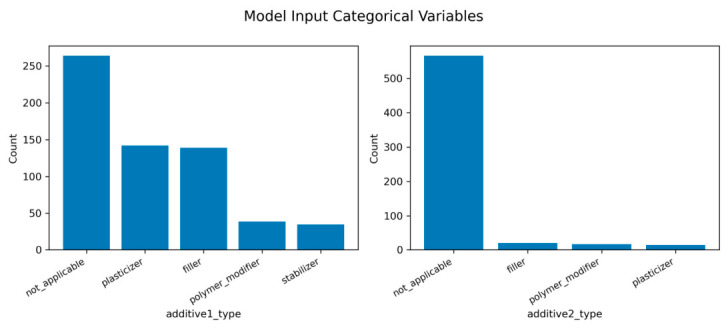
Distribution of model input categorical variables.

Figure 4 presents the distributions of 14 numerical features, showing their range and skewness. In Figure 5, the distributions of the two categorical features are presented.

### 2.4. Thermal Properties and QSPR Model Development and Validation

The study focused on predicting the key thermal properties of the polymer, namely, Tm, Tc, and Tg. Descriptive statistics of the three thermal properties are summarized in Table 6. In each case, one property was treated as the target variable, and two different predictive models (RF and XGBoost) were developed for each thermal property. To examine the influence of thermal descriptors on model performance, a sensitivity analysis was performed before final model selection for the prediction of all three thermal properties.

More precisely, for each target, multiple feature configurations were examined by including different thermal properties as additional inputs. The basic feature set comprised seven molecular and compositional descriptors: “HB_ratio_formulation”, “HV_ratio_formulation”, “Mw”, “Additive1_percentage”, “Additive1_type”, “Additive2_percentage,” and “Additive2_type”. Since Tm, Tc, and Tg are thermodynamically interrelated properties of PHB/PHBV-based systems, the potential benefit of incorporating one or two thermal properties as additional input features was systematically investigated.

Three feature space configurations were evaluated for each target property: (1) the basic feature set alone, (2) the basic set augmented with one additional thermal descriptor, and (3) the basic set augmented with both remaining thermal descriptors. This procedure limits the practical applicability of the proposed framework but was used to obtain reliable results given the limited and heterogeneous dataset. Although the inclusion of thermal descriptors generally led to improved or comparable predictive performance, this improvement resulted in a reduction in available training data, highlighting a trade-off between model accuracy and data coverage. Note that for Tc prediction, the model with basic features yielded the best overall performance. In practice, the use of thermal descriptors may also restrict applicability in early-stage material design scenarios where such properties are not yet available. Table 7 reports the dataset size corresponding to each target value and the number of features considered in each modeling scenario.

It should be noted that the reduction from the full curated dataset of 572 instances to smaller effective dataset sizes in the final models was due to data completeness requirements since only samples with measured values for all descriptors included in a given feature space configuration could be used. Consequently, the reported model performance reflects learning from these reduced effective dataset sizes rather than from the full curated database.

After selection of the target variable and the feature space, the dataset was split into training and test sets to enable robust evaluation of model performance. Thereafter, the effect of statistically extreme values was examined. Feature-based selection was carried out using dimensionality reduction through Factor Analysis of Mixed Data (FAMD), and outlier detection was performed using the interquartile range (IQR) method [44,45]. Based on the limited size of the dataset, the effect of outlier exclusion was systematically evaluated as part of the sensitivity analysis. In each case, only a small number of samples were removed, corresponding to data points exceeding 7 times the interquartile range of the target variable. Note that data points that were likely to be statistically insignificant could still carry physically meaningful and polymer-relevant information [46,47,48].

## 3. Results and Discussion

### 3.1. Data Curation

A total of 109 peer-reviewed studies were systematically mined and integrated with an in-house experimental dataset. In this manner, a structured data library of PHB/PHBV-based materials incorporating various additives and building blocks was manually curated. The curated raw dataset used in the data preprocessing stage is publicly available in the associated data repository (AUA Zenodo repository) [32]. An overview of the compositional, molecular, physicochemical, and thermal descriptors and features that were extracted from the referenced studies is provided in Table A2 in Appendix A.2.

### 3.2. QSPR Model Performance

#### 3.2.1. Sensitivity Analysis and Optimal Model Selection

Before the selection of the final model, a systematic sensitivity analysis was conducted to identify the optimal modeling configuration for each thermal property. Two cases were investigated: the composition of the input feature space and the effect of outlier exclusion on model generalization. All configurations were evaluated using both RF and XGBoost regressors, tuned via cross-validation (five folds), and compared using cross-validated (CV) R^2^ mean and standard deviation (SD). Models exhibiting signs of overfitting (training R^2^ > 0.99) or poor generalization (CV R^2^ < 0.70) were excluded from further analysis. These thresholds were applied as dataset-specific quality control filters rather than universal statistical criteria, reflecting the heterogeneity and limited sample sizes of literature-derived polymer datasets. The cross-validated R^2^ scores for all evaluated configurations are summarized in Table 8, Table 9 and Table 10, with the selected best-performing configuration highlighted in bold. The optimal models are then presented in detail in Section 3.2.2, Section 3.2.3 and Section 3.2.4.

For Tm (Table 8), multiple configurations across both algorithms and feature spaces satisfied the selection criteria, indicating that Tm prediction is robust to different modeling choices and that outlier exclusion consistently improved cross-validation stability. In contrast, Tc and Tg showed considerably fewer eligible configurations, reflecting greater sensitivity to feature space composition and preprocessing strategy; see Table 9 and Table 10, respectively. For Tc, only two configurations met the criteria, with the best performance achieved by XGBoost using the basic feature set alone, suggesting that Tc is best predicted from molecular descriptors without additional thermal inputs. For Tg, RF with all features and outlier exclusion yielded the best result. The diversity of the selected models—two RF and one XGBoost, two configurations with two thermal descriptors and one with basic features alone—reflects the target-dependent nature of thermal property prediction in PHB/PHBV systems and underlines the importance of systematic sensitivity analysis before final model selection. In the case of predicting Tg and Tm, models using only compositional features (basic set) represented a more realistic scenario for new or unseen samples, but performance improved when other thermal properties were included as inputs. This did not indicate classical data leakage but rather learning driven by strong relationships between thermal properties of polymers. A restriction is that these additional properties are often unknown in practice, reflecting real-world prediction settings. However, expanding and improving the dataset is expected to further enhance model performance and robustness, even without relying on additional thermal input features.

#### 3.2.2. Tm Prediction Model Performance

The optimal Tm prediction model was identified as the tuned RF regressor trained on the basic feature set augmented with Tc and Tg as additional thermal descriptors, with outlier exclusion applied to the training set. The nine features used for model development are summarized in the supplementary data in Table 11. The dataset comprised 118 samples and was split into training and test sets using an 80/20 ratio, resulting in 94 samples for training and 24 samples for testing.

The optimal hyperparameter values obtained via cross-validated (cv) grid search are reported in Table 12. The performance of the tuned model is reported in Table 13.

This configuration achieved a cross-validated R^2^ of 0.817 and a training R^2^ of 0.935, representing the most favorable balance between predictive performance and generalization stability among all evaluated Tm models. The relatively modest train–CV gap, combined with the low CV standard deviation, confirmed that this configuration was the most robust and reliable for Tm prediction within the constraints of the available dataset.

**Figure 6:**
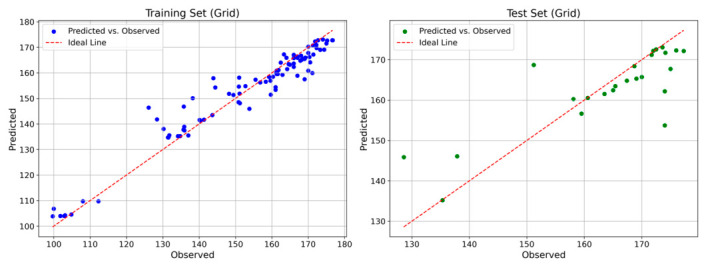
Comparison between predicted and true Tm values for the training set (**left**) and the test set (**right**) using the RF model.

A comparison of the predicted and the true Tm for the tuned RF model is presented in Figure 6.

**Figure 7:**
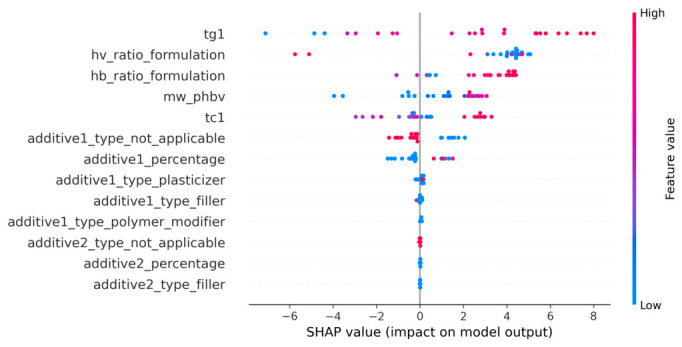
Feature importance of the RF model using SHAP values.

SHAP analysis was further undertaken to assess feature importance by examining how much each input variable contributed to the model’s performance. The resulting SHAP summary plots (Figure 7) highlighted a global view of feature effects, revealing both the magnitude and direction of each feature’s influence on the model output. The synthesis of the results indicated that the most influential features identified by SHAP analysis were associated with the formation of crystals and stability of semicrystalline polymers. In particular, higher HV content was related to lower Tm values. This trend is consistent with the literature reporting that HV incorporation can disrupt chain regularity and reduce crystallinity in PHB/PHBV systems. Similarly, lower Mw was associated with reduced Tm values, which may reflect the effect of shorter chain length and a higher density of chain ends on crystal stability.

However, with the Tc values, a higher value may reflect the fact that thicker and more perfect crystals were created, leading to higher Tm. Tg was also positively associated with Tm. This behavior may be explained by the reduced mobility of the amorphous phase of the polymers, which has been reported to promote crystal formation and a higher Tm point, as more energy is required to melt the ordered crystalline structures. The analysis further indicated that the type of additive and its percentage were also important features in the prediction of Tm. This observation may reflect the influence of additives on the crystallization behavior and chain mobility of the final biopolymer formulation and its Tm point. However, these effects depend on the nature and function of each additive used.

#### 3.2.3. Tc Prediction Model Performance

Based on the results, the best Tc prediction model was the tuned XGBoost regressor trained on the basic physicochemical and compositional feature set alone without outlier exclusion; see Table 14. The dataset consisted of 201 samples and was split into a training set with 160 samples and a test set with 41 samples using an 80/20 ratio.

The hyperparameters used in the tuned model are summarized in Table 15.

This configuration achieved a cross-validated R^2^ of 0.762 ± 0.054 and a training R^2^ of 0.972, as shown in Table 16. Closer inspection indicated that the absence of additional thermal descriptors in the optimal Tc feature space had a significant impact, unlike Tm and Tg, because crystallization temperature was a kinetic parameter influenced by the interplay between chain mobility and nucleation, which was partially reflected in the formulation descriptors such as Hv content and Mw. However, the integration of additional thermal qualities, such as input features, did not impact Tc prediction, while in several cases, it increased the variance, consistent with the physical interpretation.

**Figure 8:**
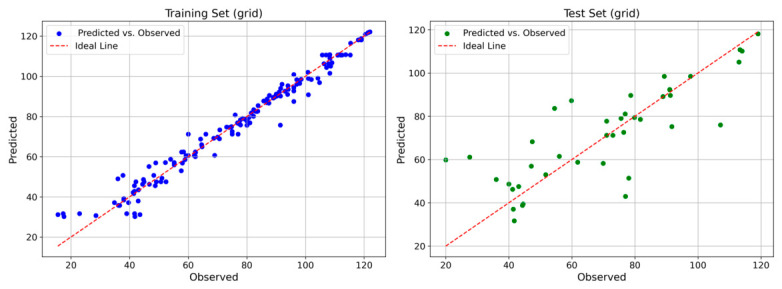
Comparison between predicted and true Tc values for the training set (**left**) and the test set (**right**) using the XGBoost model.

A comparison between the predicted and the true Tc is presented in Figure 8.

**Figure 9:**
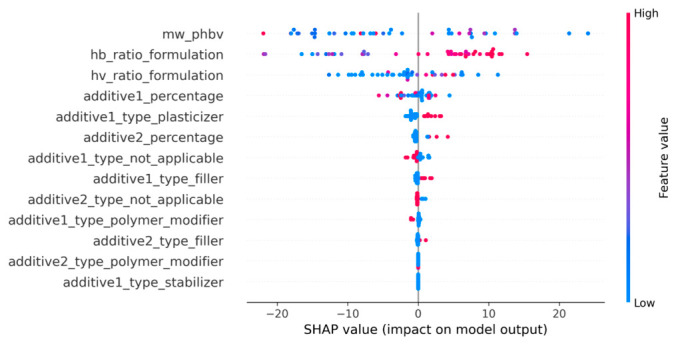
Feature importance of the XGBoost model using SHAP values.

The SHAP summary plot for the XGBoost model is presented in Figure 9. Based on the results, “Mw_PHBV” had the highest impact on model output, followed by “HB_ratio,” “HV_ratio,” and additive1 percentage. The results suggested that these features were relevant to the prediction of Tc, which is commonly associated in the literature with chain mobility, regularity, and intermolecular interactions. As such, higher Mw values may be correlated with reduced chain mobility due to higher entanglements and longer polymer chains that hindered the formation of crystals and decreased Tc values. The same effect on the prediction of Tc was observed when HV content was increased, which may be linked with the fact that crystallization became more difficult due to disrupted chain regularity. High HB monomer content may also reflect the dependence between the formation of more stable and organized crystals in the polymer matrix and higher structural rigidity, leading to higher Tc predictions. Although SHAP values represent feature importance and statistical associations within the model, the results indicated reliable QSPR performance, as the identified features are consistent with known factors influencing Tc.

#### 3.2.4. Tg Prediction Model Performance

The results indicated that the optimal Tg prediction model was the tuned Random Forest regressor trained on the basic feature set augmented with both Tm and Tc as additional thermal descriptors, with outlier exclusion applied to the training set. The features employed in the Tg model are listed in Table 17. The dataset comprised 118 samples for the Tm model since the same feature space was used.

The hyperparameter values selected for the Tg model are presented in Table 18.

This configuration achieved a cross-validated R^2^ of 0.765 and a training R^2^ of 0.960 (Table 19). The analysis indicated that including the remaining thermal properties as input features reflected the well-established thermodynamic interdependencies governing the glass transition behavior in semicrystalline polymers. Tg was influenced by chain mobility and free volume, which were directly linked to the degree of crystallinity encoded in Tc and the melting behavior captured by Tm.

**Figure 10:**
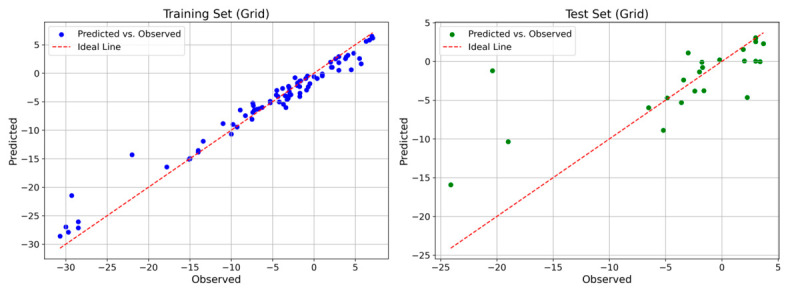
Comparison between predicted and true Tg values for the training set (**left**) and the test set (**right**) using the RF model.

A comparison between the predicted and true Tg values of the tuned model is presented in Figure 10.

**Figure 11:**
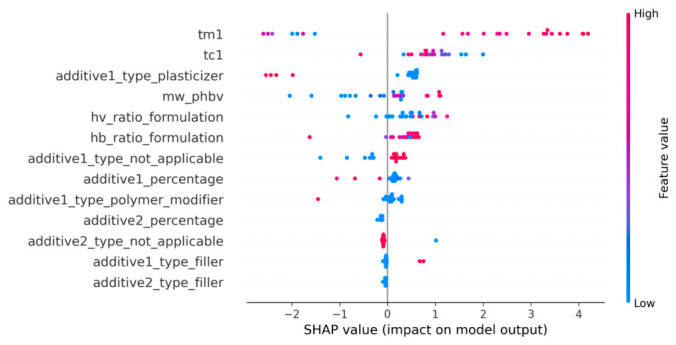
Feature importance of the RF model using SHAP values.

The SHAP analysis (Figure 11) indicated that the most influential features of the RF model included Tm, Tc, Mw, and the HV_ratio formulation. Upon closer inspection, the analysis showed that the glass transition temperature (Tg) reflected how easily polymer chains moved in the amorphous phase. Specifically, samples with higher Tm and Tc values tended to be associated with higher predicted Tg values. This association may reflect the relationship between stronger intermolecular interactions, greater chain regularity, and the development of crystalline structures, which are often linked to restricted molecular motion in amorphous regions. Similarly, the high Tg values arising from the reduction in chain mobility in the amorphous phase can be explained by high Mw values that led to an increase in chain entanglement. In contrast, an increasing HV_ratio was associated with lower predicted Tg values. This trend is consistent with reports that HV units can increase chain irregularity, reduce crystallinity, and enhance chain flexibility. In summary, the identified feature importance and the observed patterns suggest that the model captures relationships that are physically meaningful and relevant to chain mobility in the amorphous phase.

### 3.3. Comparison of Predictive Performance Across Thermal Properties

The synthesis of the predictive performance across the three thermal properties showed similarities and significant differences between the targets and optimal configurations. Applying systematic sensitivity analysis of feature space composition and outlier exclusion effects also helped identify the best-performing configuration for each thermal property. The results showed that the Random Forest algorithm was the most optimal for both Tm and Tg prediction, while XGBoost proved preferable for Tc, reflecting the target-dependent nature of thermal property modeling in PHB/PHBV systems.

All three optimal models achieved training R^2^ values below 0.990, confirming that the selected configurations avoided severe overfitting while retaining sufficient model complexity to capture nonlinear structure–property relationships. Further, the generalization performance varied by target: the Tm model achieved the highest cross-validated R^2^ of 0.817, followed by Tg at 0.765 and Tc at 0.762. The high scores underlined the generalizability of the ML models adopted in the research. As such, XGBoost and RFs could be applied across different datasets to predict the thermal features of scl-PHAs. Despite ranking third in terms of CV R^2^, the Tc model achieved the lowest cross-validation standard deviation among the three properties, indicating particularly stable generalization despite relying exclusively on the basic feature set without outlier exclusion.

The Tg model exhibited the lowest absolute error metrics among the three properties, with a cross-validated RMSE of 4.05 °C and an MAE of 2.68 °C. This outcome reflects both the performance of the model and the narrower range and lower variability in Tg values in the dataset, which naturally leads to smaller absolute errors independent of predictive strength. In contrast, the Tc model showed higher absolute errors but demonstrated the most stable generalization behavior across folds. Overall, these results highlight that predictive performance should be interpreted jointly with dataset-dependent property distributions, as error magnitudes alone may not directly reflect model quality across different thermal properties.

Feature importance analysis via SHAP revealed consistent trends: compositional descriptors such as polymer Mw, Hb, and Hv formulation ratios were among the most influential features across all three thermal properties. These findings suggested that the models assigned higher importance to variables that are known to influence the thermal behavior of PHB/PHBV systems. The SHAP trends generally agree with relationships reported in the literature, but they should be viewed as model-based associations and measures of feature importance rather than a physically direct interpretation of the features. Further insights are expected to emerge as the dataset grows and more data become available.

Nevertheless, this study has some limitations that should be considered. The thermal property data were collected from different literature sources, where experimental conditions of DSC and definitions of transition temperatures (e.g., onset, midpoint, or peak) can lead to significant shifts in the reported thermal transition temperatures. Since processing and cooling conditions can significantly affect Tc, their absence from the dataset may further limit the physical meaning of the Tc model. Additionally, characterization protocols of compositional information were not consistently reported or standardized, leading to heterogeneity in the dataset, including differences in sample preparation histories and molecular weight determination methods. In some cases, values had to be manually extracted from published graphs using PlotDigitizer, which may introduce additional noise and uncertainty.

Also, a recurring limitation across all three models was the constrained dataset size, comprising between 118 and 201 samples based on the target property. This challenge was inherent to the field of polymer informatics, where experimental thermal characterization was resource-intensive and published datasets were sparser compared to other materials domains.

For Tg and Tm prediction, models based only on compositional features represent a more realistic scenario for predicting the properties of new materials. Although better performance was achieved when other thermal properties were included as inputs, this is mainly due to the strong relationships between thermal properties rather than data leakage. However, such information is often unavailable for unknown samples.

Despite these restrictions, this work represents a first step toward a data-driven framework for predicting PHA thermal properties. Future studies with larger datasets and more standardized experimental data and testing conditions are expected to further improve model accuracy and robustness. Further insights showed that the study was also limited as it compared only XGBoost and RF models. In future work, there is a need to consider broader ML models and compare their performance in predicting the thermal features of scl-PHAs.

### 3.4. Model Validation, Generalization and Applicability Domain Analysis

#### 3.4.1. Study-Wise Splitting and External Validation

To evaluate generalization across independent studies, a source-wise (leave-one-study-out) splitting strategy was initially applied. However, due to the limited number of available studies in several test folds, this approach resulted in highly unstable performance estimates. Although cross-validation performance remained relatively high (CV R^2^ ≈ 0.73–0.78), test set performance showed large variability and, in several cases, negative R^2^ values. These results highlight the limitations of strict study-wise splitting under extreme data scarcity rather than intrinsic model failure. In particular, the imbalance in study sizes and the heterogeneity in experimental conditions led to test sets that were not statistically representative of the training distribution.

To address this constraint, an alternative external validation strategy was introduced based on chemically meaningful hold-out sets, where selected high-temperature observations from studies containing more than five instances were excluded from training. This approach resulted in more balanced and interpretable evaluation of generalization performance. Pearson correlation is particularly advantageous for evaluating unseen instances, as it captures the strength of the linear relationship between predicted and experimental values independent of scale and systematic bias.

**Figure 12:**
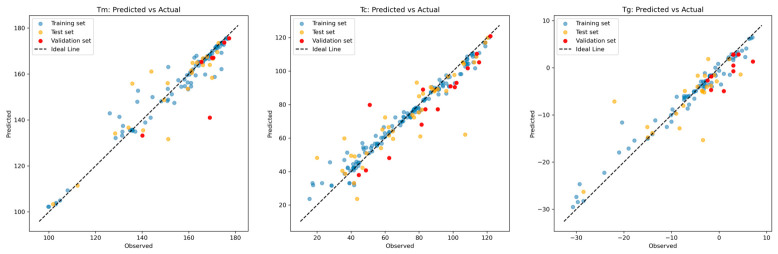
Comparison between predicted and true thermal values for the training, test and external validation sets.

Under this scheme, the models achieved improved and more stable predictive behavior, with Pearson correlation coefficients ranging from 0.71 to 0.91 across properties, as shown in Table 20. Figure 12 shows parity plots for training, test, and external validation splits. Nevertheless, validation against fully independent literature sources remains an important direction for further research.

#### 3.4.2. Effect of Molecular Weight Reconstruction on Model Performance

To evaluate the influence of molecular weight reconstruction on predictive performance, additional models were trained using datasets in which all reconstructed molecular weight descriptors were excluded. In these cases, the effective dataset size was substantially reduced; note that for Tm and Tg, the instances were fewer than 100. Across all thermal properties and model types, the resulting models exhibited mean cross-validation R^2^ values below 0.60, accompanied by large variance (>0.15) across folds.

This degradation in cross-validated performance is attributed mainly to the reduced datasets, amplifying the impact of individual studies and experimental conditions. Although reconstruction introduces approximate values, the resulting models consistently achieved higher and more stable cross-validated performance, indicating an improved bias–variance balance. These results highlight that, for sparse polymer datasets, reconstruction is essential for obtaining statistically robust and generalizable models. However, this procedure may introduce additional uncertainty, which should be taken into account when interpreting the results.

#### 3.4.3. Baseline Models and Linearity Assessment

To provide a baseline for performance comparison, several commonly used linear and kernel-based regression models were evaluated, including ordinary linear regression (LR), Ridge regression, Lasso regression, and support vector regression (SVR). These models were trained and validated using the same descriptors, data splits, and cross-validation protocol employed for the ensemble models.

Across all three target properties, linear models exhibited limited predictive capability, with mean cross-validation R^2^ values of 0.67, 0.34 and 0.38 for Tm, Tc and Tg, respectively. Regularization through Ridge and Lasso did not yield substantial improvements, indicating that the observed limitations were not due to overfitting or multicollinearity but rather to an inability of linear formulations to capture the underlying structure–property relationships.

SVR models showed improved performance relative to linear regressions, achieving cross-validation R^2^ values of approximately 0.80 for Tm (acceptable) and around 0.51–0.57 for Tc and Tg. However, SVR performance remained consistently poorer than that of the tree-based ensemble models, particularly in terms of robustness and variance across folds. The superior performance of RF and XGBoost indicated that the relationships between molecular descriptors, molecular weight characteristics, and thermal properties of PHB and PHBV were inherently nonlinear and involved higher-order feature interactions that cannot be adequately represented by linear models.

#### 3.4.4. Effect of Data Splitting Strategy

To assess whether the use of random train/test splitting leads to optimistic performance estimates, an additional evaluation was performed using a Kennard–Stone (KS) algorithm [49] to generate a more uniform and space-filling partition of the descriptor space. This approach is commonly used to mitigate sampling bias in small, heterogeneous datasets and provides a more stringent test of model robustness compared to random splitting.

Across all three thermal properties, the KS-based results confirmed that the predictive performance of the models was generally stable with respect to the splitting strategy, as shown in Table 21. For Tm and Tg, model performance remained comparable to that obtained using random splitting, indicating that the originally reported results were not artificially inflated. In contrast, Tc exhibited a moderate decrease in predictive performance under the KS split, suggesting a higher sensitivity to data heterogeneity and partitioning effects for this property. The observed differences were property-dependent rather than systematic, indicating that model performance was not primarily driven by sampling bias.

#### 3.4.5. Domain of Applicability for Model Prediction

The applicability domain (DoA) of the developed models was defined using the observed ranges of the numerical input descriptors in the training dataset (Table 22). This analysis was performed to define the regions of chemical spaces where the developed models provide trustworthy predictions, whereas outside these ranges, predictions should be treated with caution since they correspond to extrapolation.

## 4. Conclusions

This study demonstrated the successful development of an ML framework for predicting thermal properties of PHB- and PHBV-based materials. After a literature search, 109 relevant studies were selected, and their findings were integrated with an in-house experimental package to facilitate the construction of a raw dataset, comprising a total of 572 instances. The curated version of the dataset was employed for ML modeling, enabling reliable model development while minimizing overfitting and capturing nonlinear structure–property relationships, using 118 data points for Tg, 201 data points for Tc, and 118 data points for Tm. This reduction reflects the requirement that only instances with complete descriptor information have been used for model training and evaluation, and the reported model performance therefore reflects learning from these reduced datasets.

ML QSPR models based on RF and XGBoost were developed, tuned, and validated for the prediction of the three thermal properties. Following hyperparameter optimization, the best-performing configurations achieved cross-validated R^2^ values of 0.817, 0.762, and 0.765 for Tm, Tc, and Tg, respectively. RF outperformed XGBoost for Tm and Tg prediction, while XGBoost proved preferable for Tc. To examine generalization, a chemically informed external validation strategy was introduced. By holding out high-temperature data points from studies with sufficient sample sizes, an alternative split was performed. Under this setting, all thermal models achieved stable predictive behavior with strong Pearson correlation coefficients and consistent error metrics on unseen data.

Beyond predictive performance, the models provided interpretable structure–property insights and highlighted the importance of data quality and descriptor selection in polymer informatics. When data was limited and the database was relatively small, the combination of balanced performance metrics and explainability tools such as SHAP provided a practical, balanced, and effective approach for ML modeling.

Integrating datasets with interpretable ML approaches provided physical insight, supporting rational design of sustainable polymers with tailored properties, while reducing experimental time and cost and enabling application-specific optimization. In future work, the expansion of the dataset, incorporation of more detailed molecular and processing descriptors, and evaluation of alternative ML models will further validate QSPR predictive robustness. Larger datasets will enhance the predictive capability and capture more accurately the physical meaning of the features selected, revealing the importance of open-access and organized data libraries following FAIR data principles across the scientific community. The adoption of standardized testing and characterization protocols, including DSC procedures, experimental conditions, molecular weight determination and monomer composition characterization methods, would improve data consistency and comparability across studies, reducing dataset heterogeneity and further strengthening predictive performance.

## Figures and Tables

**Table 1:** Overview of worksheets and parameters in the thermal properties data libraries of scl-PHAs (PHB/PHBV formulations).

Worksheets	Parameters
Worksheet_1_Physicochemical Information (included information regarding the physicochemical properties and composition of the biopolymer)	Percentage of the PHB, percentage of the monomer PHV, weight-average molecular weight (M_w_), number-average molecular weight (M_n_), density of the polymer, PDI, the existence of an additive, the additive name, the percentage of the additive used, etc.
Worksheet_2_ Thermal properties (included information on the thermal behavior and characteristics of the polymer)	Glass transition temperature (T_g_), crystallization temperature (T_c_), melting temperature (T_m_), decomposition temperature at 5% weight loss (T_d5%_), degradation temperature (T_deg_), heat of melting fusion (ΔH_m_), heat of crystallization fusion (ΔH_c_), crystallinity (X_c %_), etc.
Worksheet_3_Metadata (contained information about the title and DOI of each study that was studied)	Study_id, author, date, title, Doi

**Table 2:** Molecular weight reconstruction.

Column	Initial Count (%)	Final Count (%)
Mw_PHBV	332 (58.0%)	367 (64.2%)
Mn_PHBV	157 (27.4%)	235 (41.1%)
PDI	180 (31.5%)	231 (40.4%)

**Table 3:** Category grouping of the feature “Additive_type_1”.

Original Category	Count	Merged Category	Count
not_applicable	247	not_applicable	247
plasticizer	79	plasticizer	115
plastisizer	36	plasticizer	
filler	61	filler	138
reinforcement	59	filler	
impact_modifier	2	filler	
nucleating_agent	16	filler	
crosslinking_agent	9	polymer_modifier	37
compatibilizer	28	polymer_modifier	
antioxidant	32	stabilizer	35
stabilizer	2	stabilizer	
antimicrobial	1	stabilizer	

**Table 4:** Category grouping of the feature “Additive_type_2”.

Original Category	Count	Merged Category	Count
not_applicable	519	not_applicable	519
plasticizer	3	plasticizer	15
plastisizer	12	plasticizer	
filler	4	filler	21
reinforcement	17	filler	
crosslinking_agent	8	polymer_modifier	17
compatibilizer	2	polymer_modifier	
blend	1	polymer_modifier	
antioxidant	6	polymer_modifier	

**Table 5:** Data type and completeness summary of the cleaned merged datasets.

Column	Count	Completeness	Dtype
Study_id	572	100.0%	int64
Instance	572	100.0%	int64
HB_ratio_formulation	567	99.1%	float64
HV_ratio_rofmulation	567	99.1%	float64
Mw_PHBV	367	64.2%	float64
Mn_PHBV	235	41.1%	float64
PDI	231	40.4%	float64
Additive1_type	572	100.0%	object
Additive1_percentage	572	100.0%	float64
Additive2_type	572	100.0%	object
Additive2_percentage	572	100.0%	float64
Tg1	273	47.8%	float64
Tc1	326	57.0%	float64
Tm1	541	94.6%	float64
Tm2	193	33.7%	float64
Td5	207	36.2%	float64
DHm1	357	62.4%	float64
Crystallinity	339	59.3%	float64

**Table 6:** Descriptive statistics of the key thermal properties.

	Tm (°C)	Tc (°C)	Tg (°C)
**Count**	541	326	273
**Mean**	158.1	85.1	−1.9
**Min**	52.0	15.5	−48.0
**Max**	180.0	126.7	43.0
**Q_2_**	166.0	86.3	−1.6

**Table 7:** Dataset sizes for the different scenarios.

Feature Space	Target Value		
	Tm	Tc	Tg
Basic	351	201	215
Basic + 1 thermal	207 (+Tg) 196 (+Tc)	196 (+Tm) 122 (+Tg)	207 (+Tm) 122 (+Tc)
Basic + 2 thermal	118	118	118

**Table 8:** CV R^2^ scores for the different feature spaces for the Tm model.

Model	Feature Space	Outlier Exclusion	Train R^2^	Test R^2^	CV R^2^ ± SD
**RF**	Basic + Tc	No	0.949	0.742	0.731 ± 0.162
		Yes	0.968	0.715	0.754 ± 0.051
	Basic + Tg	No	0.965	0.719	0.718 ± 0.089
		Yes	0.971	0.621	0.787 ± 0.074
	**Basic + Tc + Tg**	No	0.957	0.649	0.713 ± 0.220
		**Yes**	**0.935**	**0.763**	**0.817 ± 0.073**
XGBoost	Basic + Tc	Yes	0.981	0.715	0.789 ± 0.083
	Basic + Tc + Tg	Yes	0.969	0.649	0.771 ± 0.071

**Table 9:** CV R^2^ scores for the different feature spaces for the Tc model.

Model	Feature Space	Outlier Exclusion	Train R^2^	Test R^2^	CV R^2^ ± SD
RF	Basic + Tm	Yes	0.967	0.670	0.704 ± 0.095
**XGBoost**	**Basic**	**No**	**0.972**	**0.753**	**0.762 ± 0.054**

**Table 10:** CV R^2^ scores for the different feature spaces for the Tg model.

Model	Feature Space	Outlier Exclusion	Train R^2^	Test R^2^	CV R^2^ ± SD
**RF**	**Basic + Tm + Tc**	**Yes**	**0.960**	**0.731**	**0.765 ± 0.093**
XGBoost	Basic + Tc	No	0.989	0.868	0.702 ± 0.128

**Table 11:** Input features for the Tm models.

Feature	Type	Units
Tc	Numerical	Celsius
Tg	Numerical	Celsius
HB_ratio_formulation	Numerical	-
HV_ratio_formulation	Numerical	-
Mw_PHBV	Numerical	g/mol
Addtive1_percentage	Numerical	%
Additive1_type	Categorical	-
Addtive2_percentage	Numerical	%
Additive2_type	Categorical	-

**Table 12:** Optimized hyperparameter values for the RF model.

Parameter	Best Value
max_depth	10
min_samples_leaf	2
min_samples_split	5
n_estimators	50

**Table 13:** Performance of the tuned Tm model.

Metric	Random Forest (Tuned)
Train R^2^	0.935
Test R^2^	0.763
CV Mean R^2^	0.817 ± 0.073
CV Mean RMSE	8.55 ± 1.63
CV Mean MAE	5.56 ± 0.88

**Table 14:** Input features for the Tc model.

Feature	Type	Units
HB_ratio_formulation	Numerical	-
HV_ratio_formulation	Numerical	-
Mw_PHBV	Numerical	g/mol
Addtive1_percentage	Numerical	%
Additive1_type	Categorical	
Addtive2_percentage	Numerical	%
Additive2_type	Categorical	

**Table 15:** Optimized hyperparameter values for the XGBoost model.

Parameter	Best Value
max_depth	6
colsample_bytree	0.7
learning_rate	0.05
n_estimators	300
subsample	0.8

**Table 16:** Performance of the tuned Tc model.

Metric	XGBoost (Tuned)
Train R^2^	0.972
Test R^2^	0.753
CV Mean R^2^	0.762 ± 0.054
CV Mean RMSE	13.48 ± 1.54
CV Mean MAE	9.00 ± 1.32

**Table 17:** Input features for the Tg model.

Feature	Type	Units
Tm	Numerical	Celsius
Tc	Numerical	Celsius
HB_ratio_formulation	Numerical	-
HV_ratio_formulation	Numerical	-
Mw_PHBV	Numerical	g/mol
Addtive1_percentage	Numerical	%
Additive1_type	Categorical	
Addtive2_percentage	Numerical	%
Additive2_type	Categorical	

**Table 18:** Optimized hyperparameter values for the RF model.

Parameter	Best Value
max_depth	10
min_samples_leaf	1
min_samples_split	2
n_estimators	50

**Table 19:** Performance of the tuned Tg model.

Metric	Random Forest (Tuned)
Train R^2^	0.960
Test R^2^	0.731
CV Mean R^2^	0.765 ± 0.093
CV Mean RMSE	4.05 ± 1.47
CV Mean MAE	2.68 ± 0.63

**Table 20:** Performance comparison on the validation set.

Metric	Target Value		
	Tm	Tc	Tg
Pearson r	0.801	0.907	0.708
RMSE	11.70	11.59	2.89
MAE	6.38	9.81	1.97

**Table 21:** Model performance using KS splitting.

Metric	Target Value		
	Tm	Tc	Tg
Train R^2^	0.966	0.939	0.973
Test R^2^	0.850	0.845	0.720
CV Mean R^2^	0.732 ± 0.131	0.599 ± 0.113	0.732 ± 0.188

**Table 22:** Domain of Applicability (DoA) for the thermal models.

Numerical Input Feature	Target Value		
	Tm	Tc	Tg
Hb ratio formulation (mol %)	[0, 100]	[0, 100]	[0, 100]
Hv ratio formulation (mol %)	[0, 100]	[0, 100]	[0, 100]
Mw PHBV (g/mol)	[80,509, 5,240,400]	[23,900, 5,240,400]	[80,509, 5,240,400]
Additive 1 percentage (wt%)	[0, 40]	[0, 30]	[0, 40]
Additive 2 percentage (wt%)	[0, 10]	[0, 10]	[0, 10]
Tm (°C)	-	-	[99.7, 176.7]
Tc (°C)	[15.5, 114]	-	[15.5, 114]
Tg (°C)	[−30.7, 7.1]	-	-

## Data Availability

The datasets [32] used to train and evaluate the ML models were derived from curated thermal properties data reported in the literature and compiled within the framework of this study. The implementation of the ML models, including data preprocessing, feature selection, model training, and evaluation scripts, is publicly available via GitHub at: https://github.com/FSL-AUA/Temperature-model (accessed on 24 May 2026).

## Abbreviations

The following abbreviations are used in this manuscript:

scl	short chain length
PHAs	Polyhydroxyalkanoates
Tg	glass transition temperature
Tm	melting temperature
Tc	crystallization temperature
PHB	poly(3-hydroxybutyrate)
PHBV	poly(3-hydroxybutyrate-co-3-hydroxyvalerate)
XGBoost	Extreme Gradient Boosting
Mw	weight-average molecular weight
MPs	microplastics
NPs	nanoplastics
BBpPs	biodegradable plastic products
CE	circular economy
PP	polypropylene
PET	polyethylene terephthalate
PE	polyethylene
PVC	polyvinyl chloride
mcl	medium chain length
lcl	long chain length
Tdeg	degradation temperature
ML	machine learning
PHA	Polyhydroxyalkanoate
QSPR	Quantitative Structure–Property Relationship
QSAR	Quantitative Structure–Activity Relationship
Mn	number-average molecular weight
PDI	polydispersity index
R^2^	coefficient of determination
DOA	Domain of Applicability
PRISMA	Preferred Reporting Items for Systematic Reviews and Meta-Analyses
DSC	Differential Scanning Calorimetry
DOI	Digital Object Identifier
FAIR	Findability, Accessibility, Interoperability, and Reuse
AUA	Agricultural University of Athens
T_d5%_	decomposition temperature at 5% weight loss
ΔH_m_	heat of melting fusion
ΔH_c_	heat of crystallization fusion
X_c %_	crystallinity %
GPC	Gel Permeation Chromatography
SEC	Size Exclusion Chromatography
DRI	refractive index
VS	viscometry
LS	Light scatter
PMMA	poly(methylmethacrylate)
NMR	Nuclear Magnetic Resonance
CDCl_3_	deuterated chloroform (CDCl3)
CEN	European Committee for Standardization
ISO	International Organization for Standardization
UNE	Una Norma Española
EN	European Norm
RH	Relative Humidity
FAMD	Factorial Analysis of Mixed Data
IQR	interquartile range
HB	hydroxybutyrate
HV	hydroxyvalerate
CV	cross-validation
SHAP	Shapley Additive Explanations
RF	Random Forest
SD	standard deviation

## Appendix A

### Appendix A.1

In Table A1**,** we summarize the distribution of instances per study. Note that the study IDs are non-sequential as they were extracted from a larger data library.

**Table A1:** Distribution of instances across studies.

Study ID	No. of Instances	Reference	Study ID	No. of Instances	Reference
1	1	[50]	59	4	[51]
2	1	[52]	60	2	[53]
3	3	[54]	61	1	[55]
4	1	[56]	62	3	[57]
5	2	[58]	63	4	[59]
6	1	[60]	64	13	[61]
7	1	[62]	65	9	[63]
8	2	[64]	66	4	[65]
9	2	[66]	67	10	[67]
10	1	[68]	69	4	[69]
11	4	[70]	70	7	[71]
12	2	[72]	71	7	[73]
13	20	[74]	72	8	[75]
14	4	[76]	73	6	[77]
15	5	[78]	74	7	[79]
16	5	[80]	75	11	[81]
17	2	[82]	77	9	[83]
18	14	-	78	16	[84]
19	1	[85]	79	4	[86]
20	6	[87]	80	5	[88]
21	4	[89]	81	5	[90]
22	1	[91]	82	5	[92]
23	6	[93]	83	7	[94]
24	5	[95]	84	9	[96]
28	2	[97]	85	5	-
29	1	[98]	86	3	[99]
30	1	[100]	87	22	[101]
31	9	[102]	88	5	[103]
32	1	[104]	89	4	[105]
33	7	[106]	90	3	[107]
34	8	[108]	91	4	[109]
35	12	[110]	92	5	[111]
36	9	[112]	93	8	[113]
37	3	[114]	94	7	[115]
38	5	[116]	96	2	[117]
39	1	[118]	97	2	[119]
40	3	[120]	98	2	[121]
41	5	[122]	99	6	[123]
42	10	[124]	100	4	[125]
43	6	[126]	102	5	[127]
44	7	[128]	103	4	[129]
45	3	[130]	104	2	[131]
46	6	[132]	105	4	[133]
47	5	[134]	106	4	[135]
48	6	[136]	107	11	[137]
49	5	[138]	108	6	[139]
50	5	[140]	109	3	[141]
51	6	[142]	110	4	[143]
52	4	[144]	111	6	[145]
53	5	[146]	112	5	[147]
54	5	[148]	114	5	[149]
55	4	[150]	115	7	[151]
56	7	[152]	116	3	[153]
57	7	[154]	117	1	[155]
58	6	[156]	118	2	[157]
			Total	572	

### Appendix A.2

Table A2 reports a summary of features included in the thermal properties database for PHB/PHBV-based materials, which was uploaded to the AUA Zenodo repository [32]. For each feature, the table provides the feature name as used in the dataset, a concise scientific description, the measurement unit when applicable, and the observed value range across all samples. The dataset comprises a total of 25 numerical and 12 categorical features describing material characteristics, including information about composition, additives, physicochemical, and thermal properties of PHB/PHBV-based materials. The data were obtained from references [50,51,52,53,54,55,56,57,58,59,60,61,62,63,64,65,66,67,68,69,70,71,72,73,74,75,76,77,78,79,80,81,82,83,84,85,86,87,88,89,90,91,92,93,94,95,96,97,98,99,100,101,102,103,104,105,106,107,108,109,110,111,112,113,114,115,116,117,118,119,120,121,122,123,124,125,126,127,128,129,130,131,132,133,134,135,136,137,138,139,140,141,142,143,144,145,146,147,148,149,150,151,152,153,154,155,156,157].

**Table A2:** Overview of input (feature) and output (target) variables used to construct the thermal properties data library of scl-PHAs.

Feature	Description	Units	Range	Type
Study_id	Unique identifier for study instance	None	varies	categorical
Instance	Experimental run number	None	varies	categorical
Monomer A, Monomer B	Primary monomers	None	HB, HV	categorical
3 HB_ratio	Initial 3-hydroxybutyrate ratio	mol%	0–100	numerical
3 HV_ratio	Initial 3-hydroxyvalerate ratio	mol%	0–100	numerical
HB_ratio_formulation	3-hydroxyvalerate ratio in formulation	mol%	0–100	numerical
HV_ratio_formulation	3-hydroxyvalerate ratio in formulation	mol%	0–100	numerical
Molecular Weight (Mw)	Weight-average molar mass	g/mol	23,900–4,400,000	numerical
Molecular Weight (Mn)	Number-average molar mass	g/mol	35,575–2,101,000	numerical
Polydispersity Index (PDI)	Polydispersity Index	ratio	1.06–7	numerical
Density	Density of the polymer	g/cm^3^	1.09–1.25	numerical
Density_units	Units of density	g/cm^3^	-	categorical
Additive_1, Additive_2	Existence of Additives	None	Yes/No	categorical
Additive_1 name Additive_2 name	Name of the additive(s)	None	varies	categorical
Additive_1 Percentage, Additive_2 Percentage	Additive_1 content Additive_2 content	wt%	0–100	numerical
Additive_Type	Type of additive (e.g., filler, plasticizer)	None	varies	categorical
PHB(V)_percentage	PHBV content in final formulation	%	0–100	numerical
Tg1, Tg2	Glass transition temperature	Celsius (°C)	−48 to 43	numerical
Tc1,Tc2, Tmc	Crystallization temperature	Celsius (°C)	15.5–132	numerical
Tm1, Tm2	Melting temperature	Celsius (°C)	52–180	numerical
Td5	Degradation temperature where 5% of the material’s mass has decomposed	Celsius (°C)	158–329	numerical
Tdeg	Degradation temperature	Celsius (°C)	240.4–455	numerical
Temperature_units	Units of temperature	Celsius (°C)	-	categorical
DHm1, DHm2	Enthalpy change (DH) associated with the melting process	Joule/gram (J/g)	0.5–109.3	numerical
DHc1, DHc2	Enthalpy change (DH) associated with the crystallization process	Joule/gram (J/g)	0.013–97.1	numerical
Enthalpy_units	Units of enthalpy	Joule/gram (J/g)	-	categorical
Crystallinity	Degree to which a material has a well-ordered, repeating atomic or molecular structure	%	0–100	numerical
